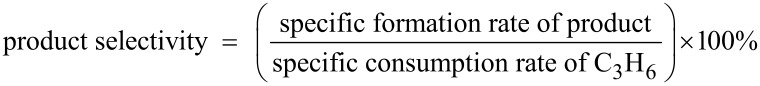# Artificial sunlight and ultraviolet light induced photo-epoxidation of propylene over V-Ti/MCM-41 photocatalyst

**DOI:** 10.3762/bjnano.5.67

**Published:** 2014-05-05

**Authors:** Van-Huy Nguyen, Shawn D Lin, Jeffrey Chi-Sheng Wu, Hsunling Bai

**Affiliations:** 1 Department of Chemical Engineering, National Taiwan University of Science and Technology, Taipei 106, Taiwan; 2 Department of Chemical Engineering, National Taiwan University, Taipei 10617, Taiwan; 3 Institute of Environmental Engineering, National Chiao Tung University, Hsin Chu 300, Taiwan

**Keywords:** artificial sunlight, light irradiation effects, photo-epoxidation, ultraviolet (UV) light, V-Ti/MCM-41 photocatalyst

## Abstract

The light irradiation parameters, including the wavelength spectrum and intensity of light source, can significantly influence a photocatalytic reaction. This study examines the propylene photo-epoxidation over V-Ti/MCM-41 photocatalyst by using artificial sunlight (Xe lamp with/without an Air Mass 1.5 Global Filter at 1.6/18.5 mW·cm^−2^) and ultraviolet light (Mercury Arc lamp with different filters in the range of 0.1–0.8 mW·cm^−2^). This is the first report of using artificial sunlight to drive the photo-epoxidation of propylene. Over V-Ti/MCM-41 photocatalyst, the propylene oxide (PO) formation rate is 193.0 and 112.1 µmol·g_cat_^−1^·h^−1^ with a PO selectivity of 35.0 and 53.7% under UV light and artificial sunlight, respectively. A normalized light utilization (NLU) index is defined and found to correlate well with the rate of both PO formation and C_3_H_6_ consumption in log–log scale. The light utilization with a mercury arc lamp is better than with a xenon lamp. The selectivity to PO remains practically unchanged with respect to NLU, suggesting that the photo-epoxidation occurs through the same mechanism under the conditions tested in this study.

## Introduction

It is agreed that light, especially its wavelength spectrum and intensity, is a crucial factor for efficient photocatalysis. A photocatalytic reaction occurs only when the illumination with light enables the generation of highly reactive species such as hydroxyl radicals (OH•) and oxy radicals (O•) [1]. The light intensity in photocatalysis has attracted considerable attention. The positive effect of increasing the light intensity on photocatalytic reactions is a common phenomenon, which has been observed in, e.g., the photo-degradation of gaseous formaldehyde [2], dye [3–5] and polychlorinated dibenzo-*p*-dioxins [6], and the disinfection of *Escherichia coli* [7–9]. How the light energy can be effectively utilized in a heterogeneous photocatalysis process is under debate, and both the intensity and the exposure time to light irradiation need to be considered [4,10]. In the photo-decomposition of organic species and the inactivation of bacteria under ultraviolet sources (UV-A and UV-C), low-intensity light with long exposure times resulted in a better light-utilization efficiency than light of high intensity with short exposure times [10]. In addition, the wavelength of the irradiation is another important factor that can affect the efficiency of photocatalysis. It is believed that the shorter wavelength of irradiation can promote the electron–hole generation and consequently enhance the efficiency of the catalyst. This has been observed in the CO_2_ photo-reduction over Ag/TiO_2_ or TiO_2_ [11], the photo-degradation of 4-chlorophenol over TiO_2_ [12] and the photo-decomposition of organic contaminants over CaBi_2_O_4_ [13]. No previous study discusses that the suitable wavelength needs to match the absorbance range of the catalyst and also needs to be energetic enough to generate active species.

Propylene oxide (PO) is an intermediate chemical widely used in the chemical industry, and its market size is predicted to have an average annual growth of 5% [14]. However, the current commercial processes to produce PO are not environmentally friendly because of the significant amounts of byproducts [15–16]. Much effort has been devoted to develop green PO production processes and photo-epoxidation utilizing light energy and O_2_ oxidant under mild conditions attracts much attention. Differently designed photocatalysts were examined [17–24], and the reaction conditions such as reaction temperature [18], light irradiation [25–26] and oxygen/propylene ratio [23,27] were also tested. Yoshida and co-workers reported the first systematic investigation of the photo-epoxidation of propylene over more than 50 silica-supported metal oxides, in which TiO*_x_*/SiO_2_ was the most effective photocatalyst [20]. Amano et al. reported that Rb-ion-modified V_2_O_5_/SiO_2_ was their best photo-epoxidation catalyst under UV-C light [24]. In our previous study, V-Ti/MCM-41 photocatalyst showed good PO yield under UV light [17]. However, the effects of light irradiation on the photo-epoxidation of propylene have not been systematically examined. Yamashita et al. carried out photo-epoxidation experiments by using a high-pressure mercury lamp equipped with 3 UV cut-filters, but they did not discuss the effect of light wavelengths [28]. Xenon lamps are the most commonly used light sources in these studies [24,29]. No previous work, to the best of our knowledge, attempts the use of artificial sunlight to drive the photocatalytic epoxidation [30].

In this study, we compared the photo-epoxidation over V-Ti/MCM-41 photocatalyst with different wavelengths (365, 320–500 and 250–400 nm) and different light intensities in the range of 0.1–0.8 mW·cm^−2^. In addition, artificial sunlight from a solar simulator with/without an Air Mass 1.5 Global (AM1.5G) filter was also used to drive the photo-epoxidation reaction. The performances of photo-epoxidation under different light sources are compared and discussed.

## Results and Discussion

### Photocatalyst characterization

**Figure 1:**
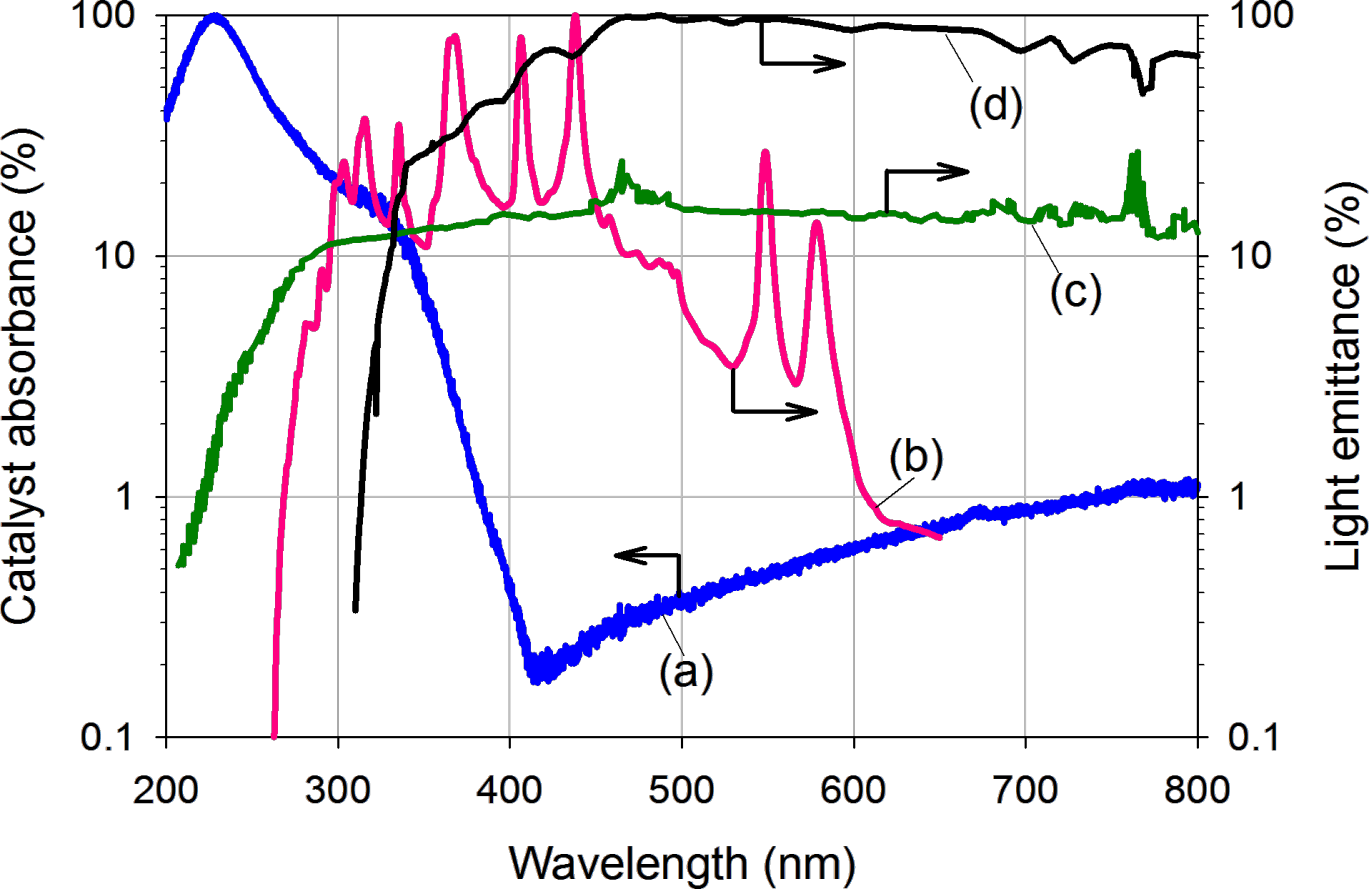
The spectrum of: (a) UV–vis absorption of V-Ti/MCM-41, and emission of (b) 200 W mercury arc lamp, (c) 300 W Xe lamp (extracted from [22]) and (d) AM1.5G filter [31].

Figure 1 compares the absorbance spectrum of the prepared photocatalyst and the light emission spectrum from a Hg arc lamp, and a Xe lamp without filter [22] and with AM1.5G filter [31], respectively. The emission spectrum from the Hg arc lamp is in the range of 260–650 nm while those spectra from Xe lamp without filter and with AM1.5G filter are 200–2400 nm and 310–2400 nm (not shown in the range of Figure 1), respectively. The UV–vis absorbance confirms the absorption band of 200–380 nm. The strong absorbance at about 220 nm is attributed to the charge transfer band of tetra-coordinated titanium in the framework while the weaker band at 340 nm is attributed to the charge transfer band of tetra-coordinated vanadium in the V^5+^ state [32]. The overlap of catalyst absorbance and light emission spectra, in the wavelength range from 260 to 380 nm, is the main contribution to the photo-reaction activity.

**Figure 2:**
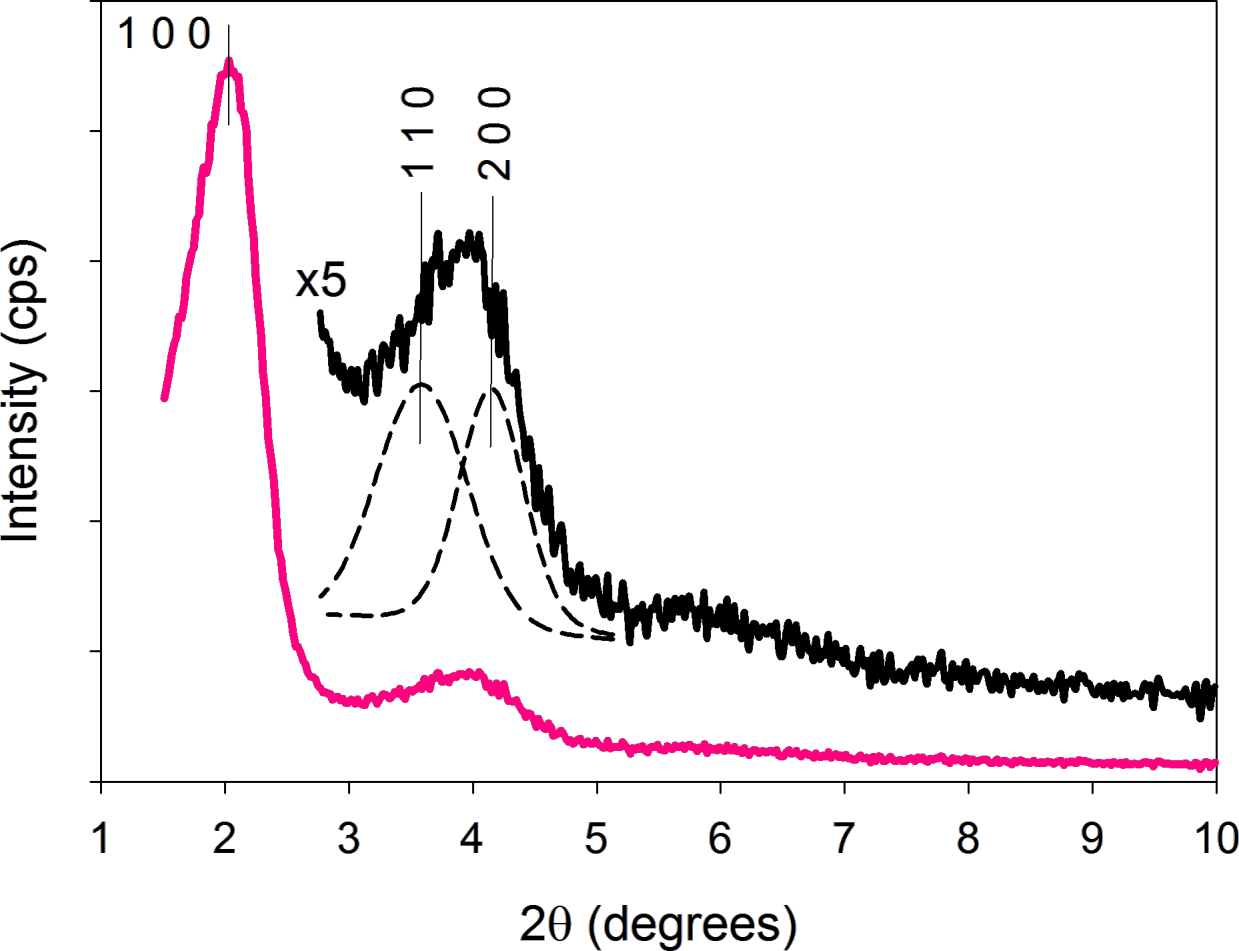
The low-angle XRD pattern of V-Ti/MCM-41.

**Figure 3:**
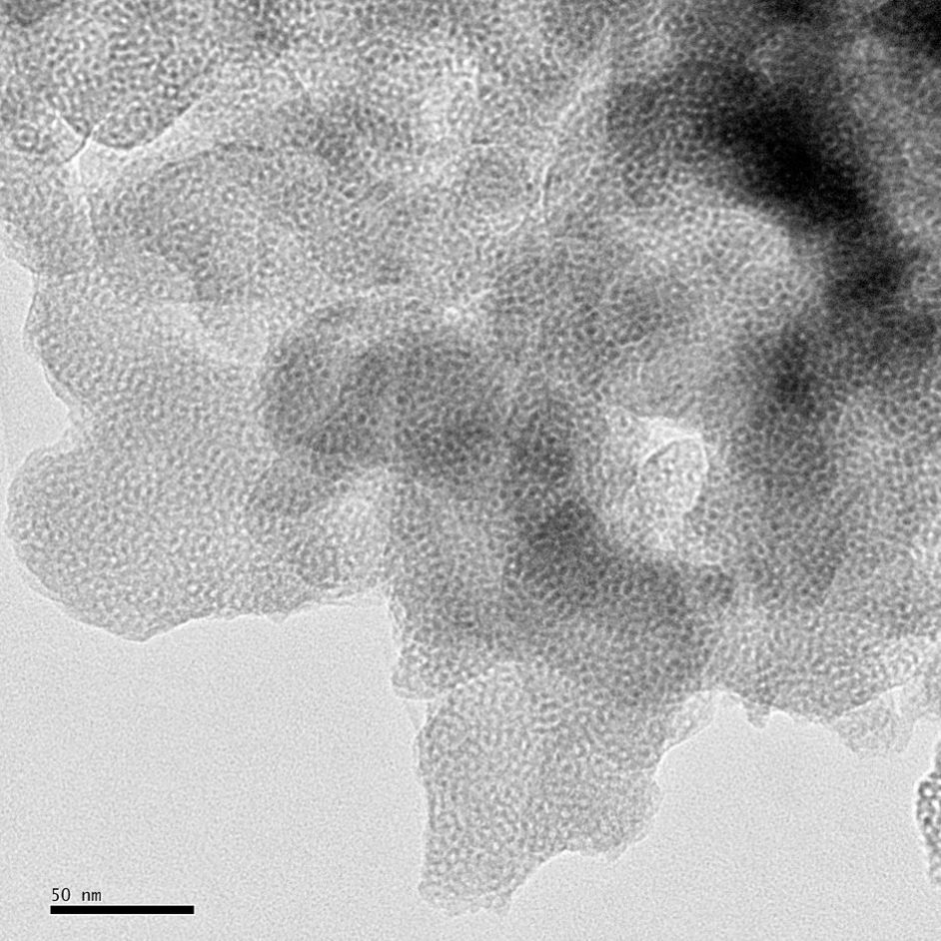
The HRTEM images of V-Ti/MCM-41 photocatalyst.

Figure 2 shows the low-angle XRD pattern of V-Ti/MCM-41 photocatalyst. The XRD pattern indicates a mesoporous hexagonal lattice with a clear feature of (100). The (110) and (200) peaks are not well-separated, maybe due to the high calcination temperature of 823 K [33]. The HRTEM image of V-Ti/MCM-41 in Figure 3 reveals a uniform hexagonal structure, which is a distinctive feature of MCM-41. The pore diameters of catalyst estimated from TEM was approximately 3 nm.

**Figure 4:**
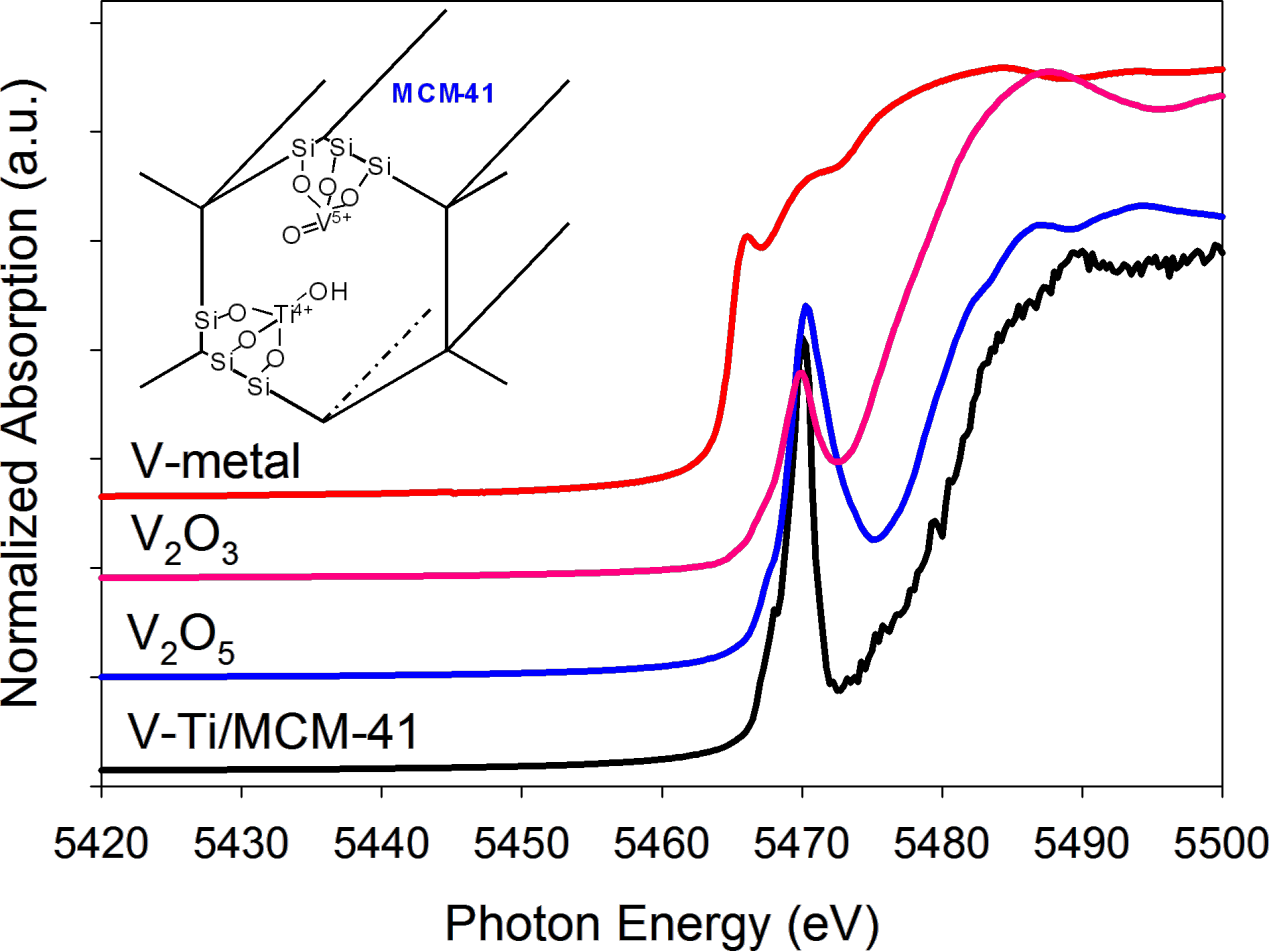
Summary of the V K-edge characterization of V-Ti/MCM-41 with references by XANES and proposed structures of V-Ti/MCM-41 (inset).

We previously proposed that the titanium in V-Ti/MCM-41 should be Ti^4+^ with a tetrahedral coordination [17]. The K-edge XANES of vanadium (Figure 4) suggests its oxidation state to be V^5+^. A possible local structure of tetra-coordinated V- and Ti-oxides in V-Ti/MCM-41 is proposed in the inset of Figure 4. Both Ti^4+^ and V^5+^ are anchored by three oxygen atoms (each linked to silicon) and with an exposed (Ti^4+^–OH) or an exposed (V^5+^–O^2−^). We expect that the design of V-Ti/MCM-41 can lead not only to the direct excitation of (Ti–O) moieties by UV irradiation but also to the indirect excitation through a charge transition from (V^4+^–O_L_^−^)* states as proposed in [34–35], which brings up a high photocatalytic activity [34]. Furthermore, Amano et al. reported that the lattice oxygen in the excited triplet state (V^4+^–O_L_^−^)* is considered to exhibit electrophilic character, which preferably attacks the double bonds in propylene [35]. Hence, the V-Ti/MCM-41 is expected to perform a selective photocatalytic epoxidation of propylene.

### Photocatalytic epoxidation of propylene

There is no activity observed if the experiment is conducted in the absence of either the photocatalyst or light irradiation. Evidently, the propylene epoxidation over V-Ti/MCM-41 is mainly photo-catalysed. Table 1 summarizes the consumption rate of C_3_H_6_, the formation rate of PO and the product selectivity under different irradiation conditions. These values are averages obtained on stream within 3–6 h of photoreaction. The major products included propylene oxide (PO), propionaldehyde (PA) and acetaldehyde (AA) while the minor products were acetone (AC) and ethanol (EtOH).

**Table 1:** Overview of the photocatalytic epoxidation of propylene.^a^

entry	light sources			C_3_H_6_ consumption rate (µmol·g^−1^·h^−1^)	PO formation rate (µmol·g^−1^·h^−1^)	selectivity (%)				
	lamp	filter	intensity (mW·cm^−2^)			AA	EtOH	PO	PA	AC

1	200 W Mercury Arc	365 nm	0.1	89.2 ± 2.2	25.8 ± 1.1	38.9	2.5	29.0	20.5	9.1
2			0.2	145.4 ± 1.8	42.9 ± 0.2	39.5	3.5	29.5	19.9	7.6
3		320–500 nm	0.1	136.3 ± 1.8	48.4 ± 1.1	30.3	ND	35.5	27.0	7.2
4			0.2	269.3 ± 11.6	114.2 ± 7.8	28.9	ND	42.3	23.7	5.1
5			0.4	276.7 ± 10.5	80.6 ± 3.2	42.7	4.5	29.1	17.5	6.8
6			0.6	285.3 ± 3.3	92.9 ± 1.6	38.9	4.4	32.6	16.9	7.2
7			0.8	340.9 ± 2.7	108.4 ± 1.2	38.8	4.7	31.8	17.4	7.3
8		250–400 nm	0.2	329.9 ± 9.3	100.5 ± 2.0	35.4	2.3	30.5	23.6	8.2
9			0.4	424.4 ± 21.0	140.6 ± 6.2	32.9	2.4	33.2	24.1	7.4
10			0.8	551.9 ± 2.4	193.0 ± 1.2	30.7	2.3	35.0	25.9	6.1
11	300 W Xe	AM1.5G	1.6	208.4 ± 14.3	112.1 ± 8.9	13.0	ND	53.7	23.1	10.3
12		—	18.5	287.3 ± 14.4	172.1 ± 8.9	15.9	ND	59.9	21.0	3.2

^a^Reaction conditions: 0.01–0.02 g photocatalyst; feed gas C_3_H_6_/O_2_/N_2_ = 1:1:16 in vol % at GHSV = 6000 h^−1^ and *T* = 312–323 K. The data were obtained on stream within a cycle (3–6 h in reaction). AA: acetaldehyde, EtOH: ethanol, PO: propylene oxide, PA: propionaldehyde, AC: acetone, and ND: not detected.

### Artificial sunlight irradiation

**Figure 5:**
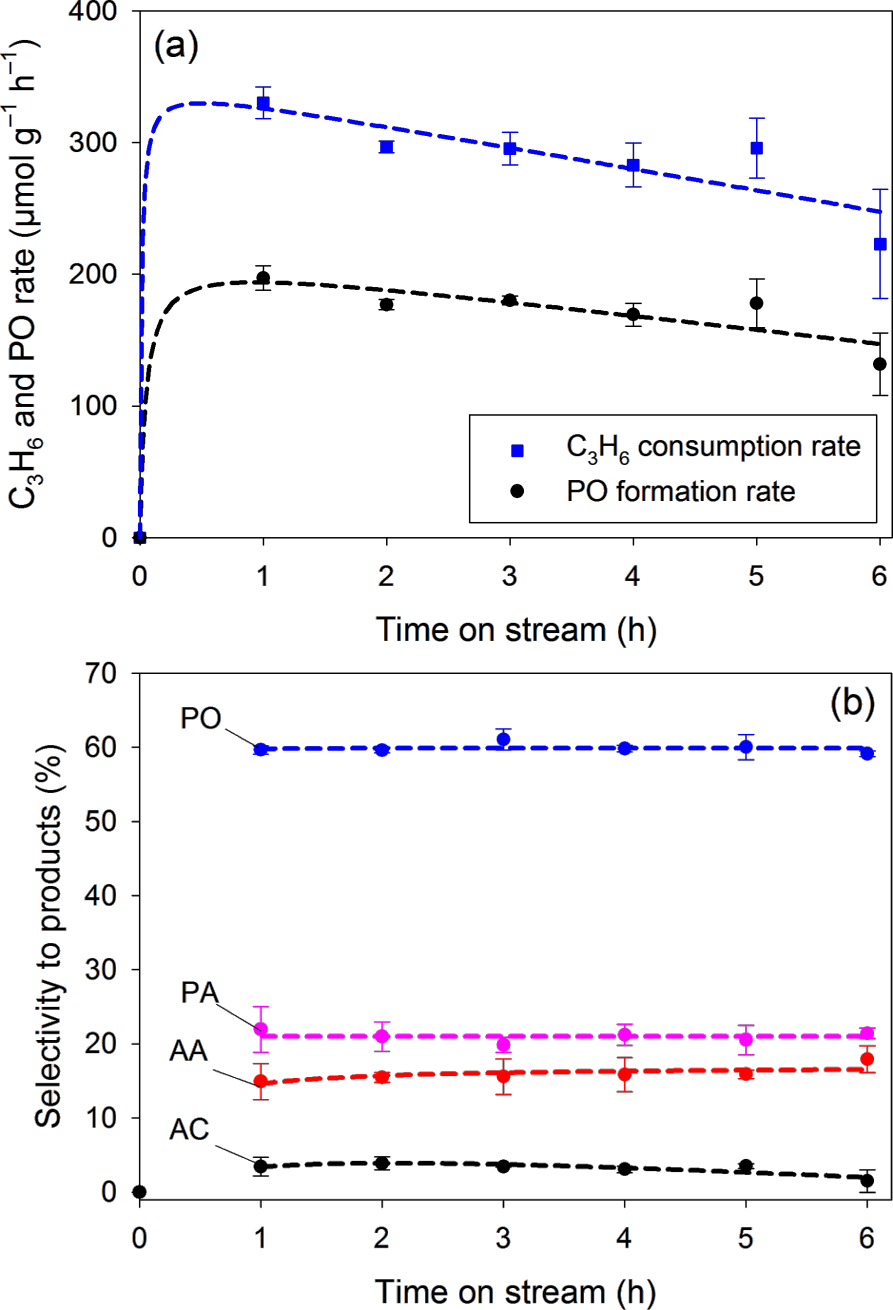
Time course of the photo-epoxidation of propylene with molecular oxygen under UV–visible light irradiation (without an AM1.5G filter; 18.5 mW·cm^−2^): (a) C_3_H_6_ consumption rate and PO formation rate, (b) Selectivity to products. Lines are presented for guiding and are not based on a kinetic model.

Recently, sun-light-driven photocatalysis has received much attention. In this section, we examined the photo-epoxidation of propylene over V-Ti/MCM-41 by utilizing UV-visible light (without an AM1.5G filter) and artificial sunlight (with an AM1.5G filter). With UV–visible light, the C_3_H_6_ consumption rate and the PO formation rate with time on stream are shown in Figure 5a. Within a 6 h test, its performance decreased monotonously with time, but the selectivity of products on stream did not change as shown in Figure 5b, which indicates PO as the dominant product with a selectivity of 59.9%. This PO selectivity is significantly higher that of experiments using only UV light.

**Figure 6:**
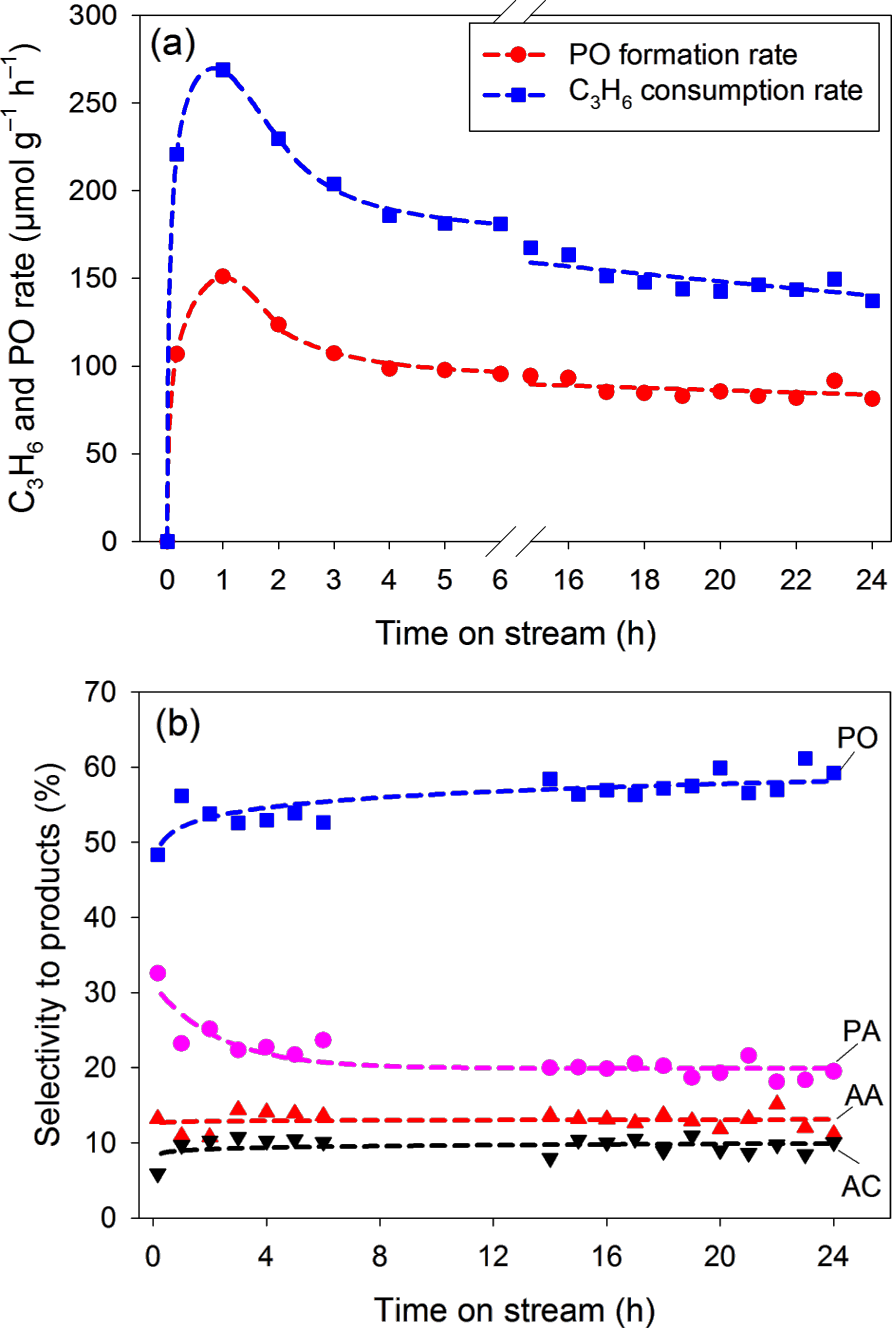
Time course of the photo-epoxidation of propylene with molecular oxygen under artificial sunlight (with an AM1.5G filter; 1.6 mW·cm^−2^): (a) C_3_H_6_ consumption rate and PO formation rate. (b) Selectivity to products. Lines are presented for guiding and are not based on a kinetic model.

Figure 6 shows the time-dependent behavior of the photocatalytic reaction when using artificial sunlight. With respect to time on stream, the PO formation rate increased to a peak value of 151 µmol·g_cat_^−1^·h^−1^ after 1 h and then it decayed to 81 µmol·g_cat_^−1^·h^−1^ after 24 h reaction time (Figure 6a). The product distribution was not significantly changed by the inclusion of the AM1.5 filter. The main product PO still maintained a selectivity of approximately 60% (Figure 6b). To the best of our knowledge, this is the first report of using artificial sunlight to drive the photocatalytic epoxidation of propylene.

Both of UV–visible light and artificial sunlight show a similar and stable product distribution (Figure 5b and Figure 6b). The use of AM1.5G filter decreased the photo-activity but similar trends in PO formation rate and C_3_H_6_ consumption rate with time on stream are also found in Figure 5a and Figure 6a. The similar product distribution implies that the reaction mechanism was not changed by the presence of the AM1.5G filter. The filter reduced the light intensity significantly from 18.5 to 1.6 mW·cm^−2^. Therefore, we believe that the decreased photo-activity when using the AM1.5 G filter is mainly attributed to the decreased light intensity.

### UV-light irradiation

**Figure 7:**
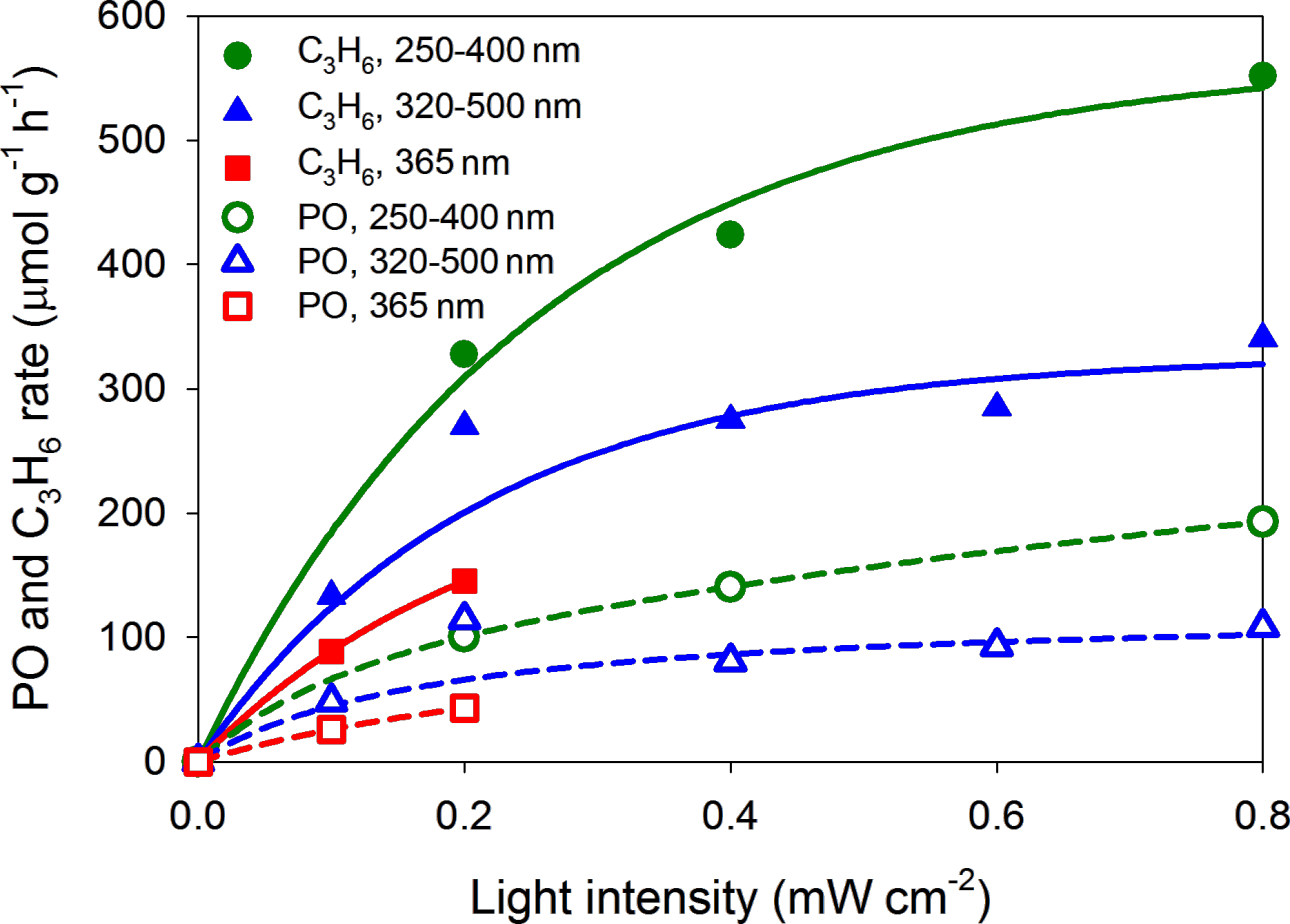
The correlation between UV-light intensity (200 W mercury arc lamp) and C_3_H_6_ consumption rate and PO formation rate. Lines are presented for guiding and are not based on a kinetic model.

Among the UV-irradiation experiments shown in Table 1, entry 4 shows a good selectivity to PO (42.3%) while entry 10 shows excellent performance of both C_3_H_6_ consumption rate and PO formation rate, 551.9 and 193.0 µmol·g_cat_^−1^·h^−1^, respectively. Figure 7 shows that the illumination of shorter wavelengths (250–400 nm) resulted in a better performance (higher C_3_H_6_ consumption rate and higher PO formation rate) than that with 365 nm or 320–500 nm illumination. In response to intensity, both C_3_H_6_ consumption rate and PO formation rate show an initial linear increase and then the increase gradually levels off at strong intensities. The nonlinear behavior can be explained by either that the effect of light scattering becomes significant or that the reactions strongly compete with processes involving electron–hole pair recombination and with those involving the participation of photo-generated holes in surface photo-reactions at high-intensity illumination [6,36–37]. On the other hand, the initial linear behavior suggests that the recombination of electron–hole pairs is negligible at low intensity illumination.

**Figure 8:**
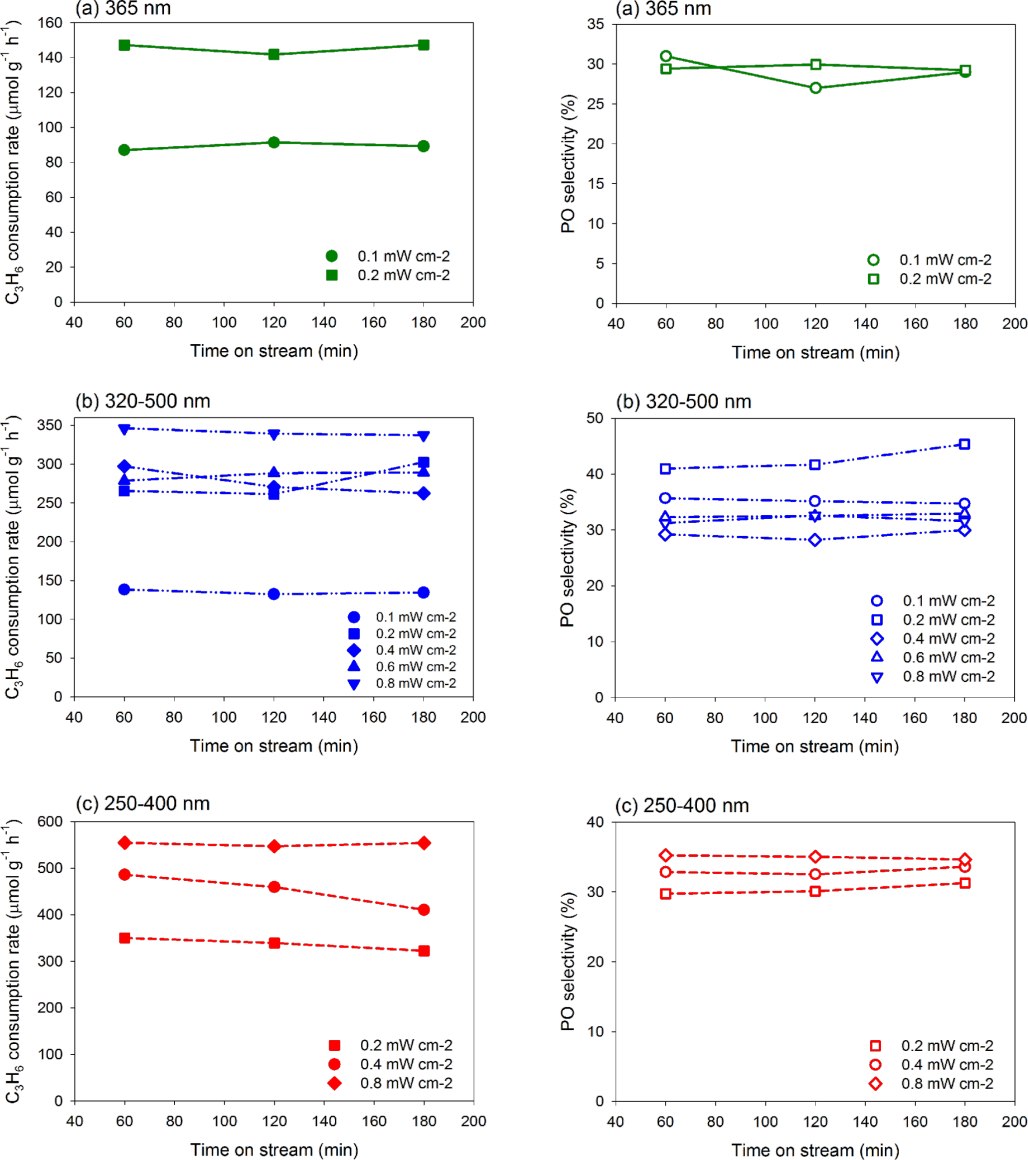
Time course of the photo-epoxidation of propylene with molecular oxygen under UV light for C_3_H_6_ consumption rate and PO selectivity under different filters conditions: (a) 365 nm, (b) 320–500 nm and 250–400 nm.

Figure 8 shows the time-dependent behavior of the photocatalytic reaction when using different filters with UV light. The PO selectivity was stable even under UV-C range of 250–400 nm. On the whole, an increase in light intensity promoted the activity and resulted in increased C_3_H_6_ consumption rate.

### Comparison of artificial sunlight and UV light irradiation

To understand how photo-epoxidation efficiency changed with wavelength, we compared the effect of photon absorption based on the spectra of different lamps and/or filters. Since not all the light delivered to the photocatalyst can be absorbed, we defined the normalized light utilization (NLU) of V-Ti/MCM-41 photocatalyst as the fraction of light that can possibly activate the photoreaction by Equation 1.

[1]
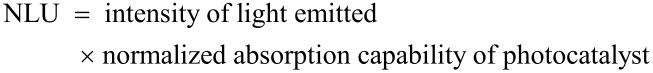


The total absorption capability of V-Ti/MCM-41 photocatalyst was calculated by integrating the normalized UV–vis spectrum from the lower cut-off wavelength of irradiation light (260 nm for UV, 200 nm for visible light and 310 nm for artificial light) to the cut-off absorbance of the catalyst (380 nm). The ratio of the integrated absorbance over the filtered range of the light source to the total absorption capacity is defined as the normalized absorption capability. Table 2 shows the calculated NLU of V-Ti/MCM-41 photocatalyst for visible light, artificial sunlight and UV light at three filtered wavelength range (365 nm, 320–500 nm and 250–400 nm).

**Table 2:** A quantitative evaluations of normalized light utilization by V-Ti/MCM-41 photocatalyst.

entry	light sources		intensity of light emitted (mW·cm^−2^)	normalized active absorbed light by catalyst^a^ (a.u.)	normalized light utilization^b^ (mW·cm^−2^)
	lamp	filter			

1	200 W mercury arc	365 nm	0.1	0.03	0.003
2			0.2		0.006
3		320–500 nm	0.1	0.21	0.021
4			0.2		0.042
5			0.4		0.084
6			0.6		0.126
7			0.8		0.168
8		250–400 nm	0.2	1.00	0.200
9			0.4		0.400
10			0.8		0.800
11	300 W Xe	AM1.5G	1.6	0.29	0.464
12		—	18.5	1.00	18.500

^a^The normalized absorption capability is the ratio between area of irradiation at different wavelength and the area of full UV–vis absorbance spectrum. ^b^Normalized light utilization was calculated directly by Equation 1.

**Figure 9:**
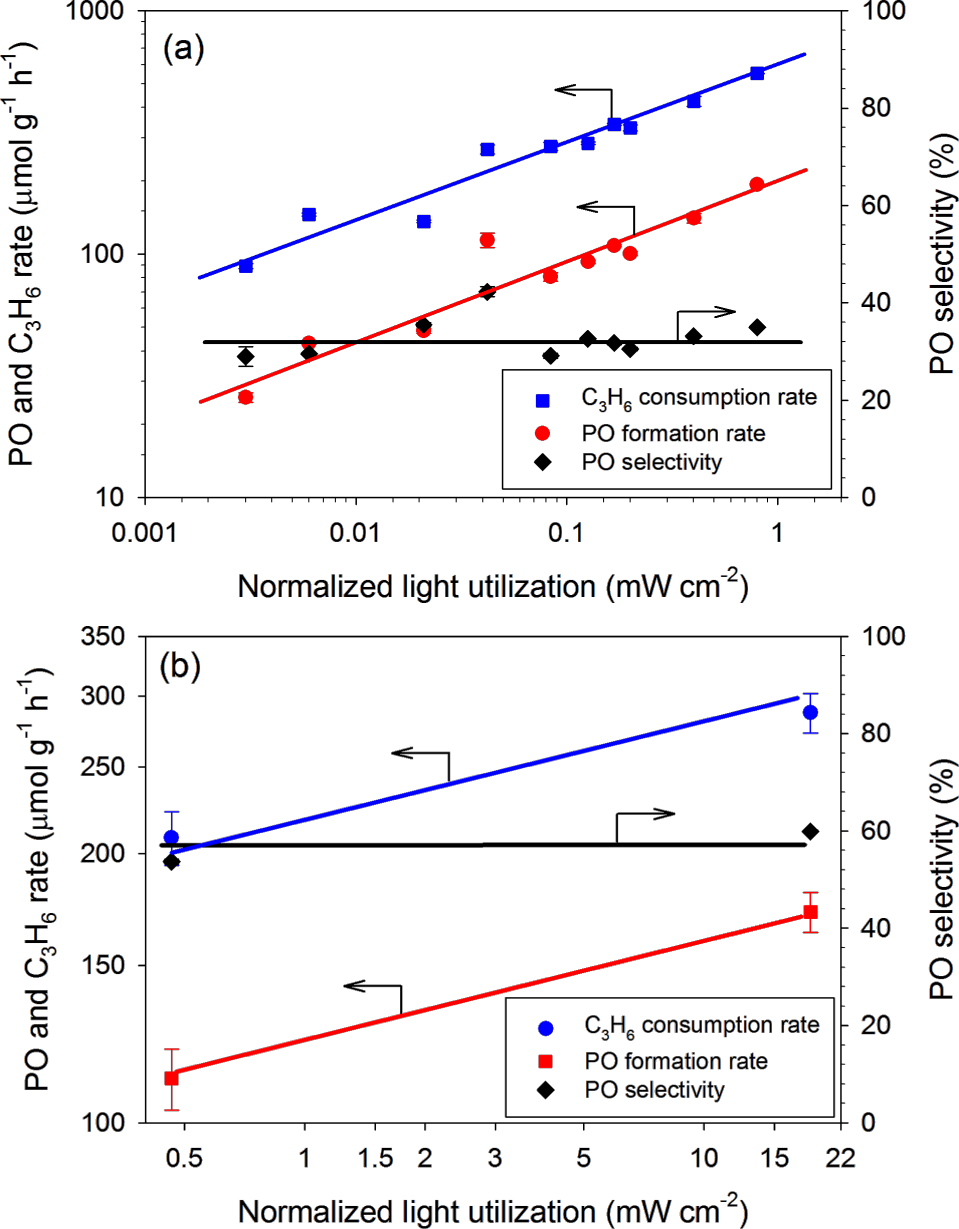
The PO formation rate, C_3_H_6_ consumption rate and PO selectivity over V-Ti/MCM-41 versus the normalized light utilization by V-Ti/MCM-41 photocatalyst over (a) a mercury arc lamp and (b) a xenon lamp (see Table 2). Lines are presented for guiding and are not based on a kinetic model.

Figure 9 shows that both C_3_H_6_ consumption rate and PO formation rate increased with the calculated NLU, regardless of the filtered wavelength range. For the UV light source (Figure 9a), it suggests that the photon flux, i.e., the light intensity, is equally efficient for the photo-epoxidation of propylene when the wavelengths of photon were filtered to 365, 350–500, or 250–400 nm range. This also implies that the energy in these three filtered ranges is sufficient to activate oxygen and/or propylene. The correlation between the rate of PO formation or of C_3_H_6_ consumption versus NLU in the log–log scale can be expressed by Equation 2 and Equation 3 as the rate expression for the UV-irradiated photo-epoxidation. The ratio of these two rate expressions indicates a constant PO selectivity of 40%.

[2]
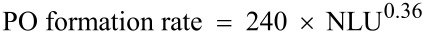


[3]
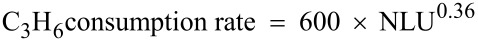


The rate expressions of both PO formation and C_3_H_6_ consumption with a Xe lamp were estimated by the data with and without AM1.5G filter as shown in Figure 9b, and the results are shown in Equation 4 and Equation 5. These rates are lower but the PO selectivity is higher than that of using mercury arc lamp.

[4]
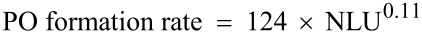


[5]
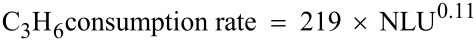


The different dependency on NLU in these rate equations of PO formation and C_3_H_6_ consumption between UV and UV–visible/artificial sunlight may be due to the difference in light wavelength and intensity. Both UV and UV-visible/artificial sunlight resulted in a nearly constant PO selectivity, regardless of the filter or the light intensity. Based on this fact, we believe that the photocatalytic epoxidation of propylene over V-Ti/MCM-41 photocatalyst occurred through the same mechanism regardless of the absorbed wavelengths within the range of study.

We have compared the C_3_H_6_ consumption rate of V-Ti/MCM-41 when using UV (0.8 mW·cm^−2^, 250–400 nm), artificial sunlight (1.6 mW·cm^−2^ when with AM1.5G filter) and UV–visible light without AM1.5G filter (18.5 mW·cm^−2^) from entry 10, 11 and 12, respectively. Although the light intensity differed strongly when comparing both UV–visible light and artificial sunlight, the C_3_H_6_ consumption rate (551.9, 287.3 and 208.4 µmol g_cat_^–1^ h^–1^, respectively) did not significantly change. A possible explanation is that the emission wavelength of UV light fits better with the spectral absorption of V-Ti/MCM-41 than the other irradiation types (Figure 1). Wendl et al. reported a similar observation when they compared the effects of various lamps [38]. The non-fitting wavelengths of the visible light source may provide an additional heating to the catalyst.

**Figure 10:**
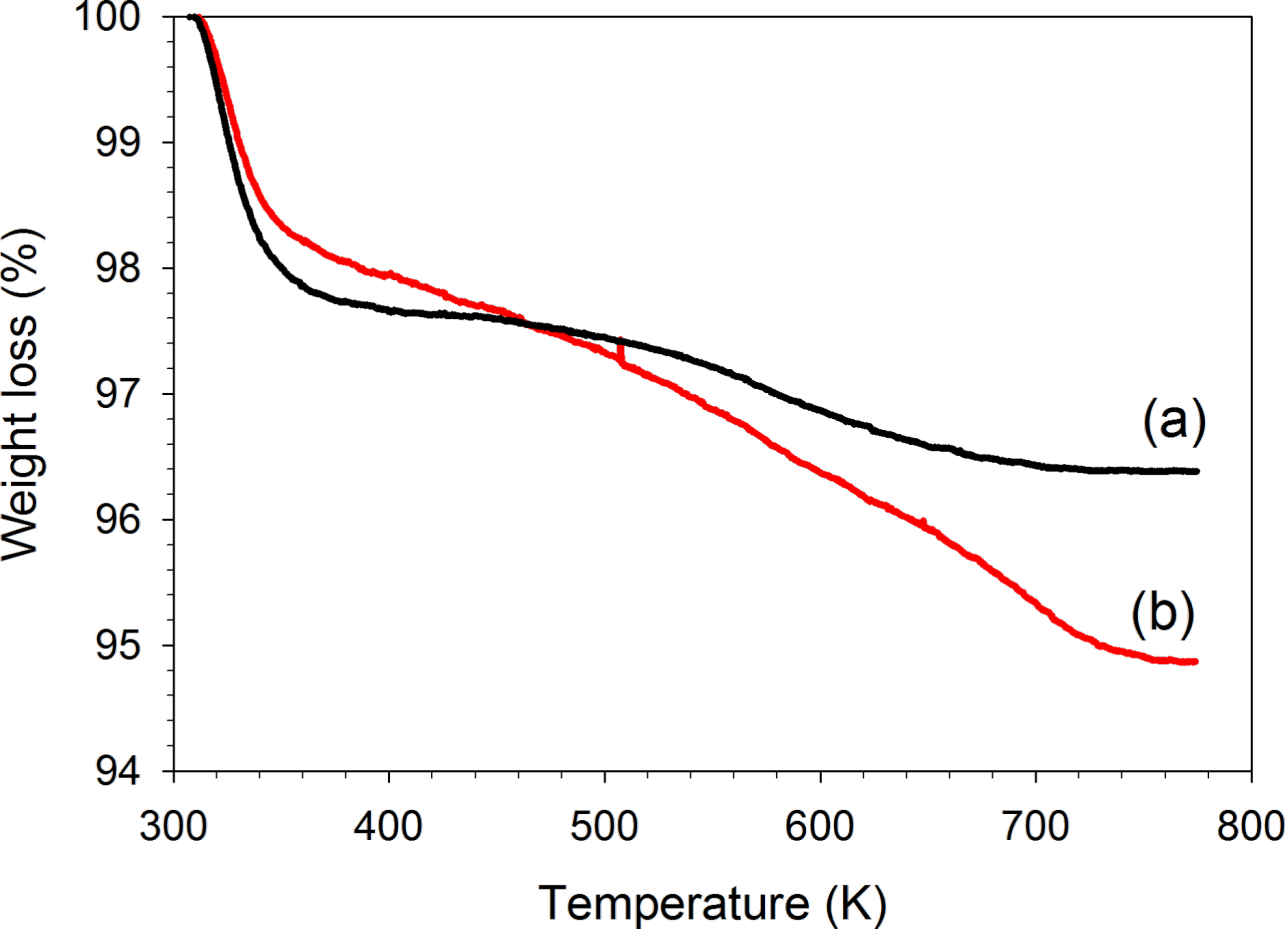
The TGA weight loss curves V-Ti/MCM-41 photocatalyst using O_2_ as the sweep gas: (a) fresh catalyst and (b) catalyst deactivated by the photo-epoxidation of propylene.

The decay in the photo-activity with time on stream may be due to fouling caused by strongly adsorbed organic species. Comparing to UV-irradiation conditions, the selectivity to AA was lower when using artificial sunlight (Table 1). Takeuchi et al. reported that products with a carbonyl group such as AA can easily adsorb on active Ti^4+^ sites [39]. Therefore, the low selectivity to AA might be due to its accumulation on catalyst surface, which can consequently cause the higher standard error on the C_3_H_6_ consumption rate observed with both UV–visible light and artificial sunlight. The condensation of AA molecules may lead to larger and heavier species such as hexa-2,4-dienal and 3-methylpentanedial [40]. The TGA weight loss curves of the spent and the fresh V-Ti/MCM-41 are compared in Figure 10. The weight loss below 400 K is attributed to the removal of adsorbed water while that above 400 K can be attributed to burn-off of the remaining organic species [41]. The different decay rates observed with UV and UV–visible light/artificial sunlight may come from the different fouling level of strongly adsorbed organic species.

### Current status of photo-epoxidation of propylene

Table 3 compares the propylene photo-epoxidation performance of this study and those reported in the literature. Entries 1–4 show that V_2_O_5_/SiO_2_ exhibits visible-light-driven photocatalytic activities when using a solar simulator; Rb ion-modified V_2_O_5_/SiO_2_ performs well with a high C_3_H_6_ consumption rate of 563.5 µmol·g_cat_^−1^·h^−1^. Rubidium ions can effectively modify isolated VO_4_ via a mono-oxo terminal oxygen, which is proposed to improve the PO formation rate [24]. The V-Ti/MCM-41 used in this study had only half of that C_3_H_6_ consumption rate (entry 5) under light from solar simulator, but both V-Ti/MCM-41 and Rb-V_2_O_5_/SiO_2_ had nearly the same PO formation rate. Although the two experiments were carried out under different conditions, both demonstrate good performance of propylene photo-epoxidation. Artificial sunlight has been successfully used and it showed interesting photo-activity (112.1 µmol·g_cat_^−1^·h^−1^ of PO formation rate). In this contribution, 193.0 µmol·g_cat_^−1^ h^−1^ (with 0.8 mW·cm^−2^ UV in the range of 250–400 nm) is the highest PO formation rate achieved among a variety of conditions.

**Table 3:** Comparison of artificial sunlight and other light sources.

entry	photocatalyst	lamp	reactants	C_3_H_6_ consumption rate (µmol·g^−1^·h^−1^)	PO formation rate (µmol·g^−1^·h^−1^)	PO selectivity (%)	ref.

1	0.18 wt % V_2_O_5_/SiO_2_	300 W Xe	C_3_H_6_/O_2_/He = 2:1:7 (GHSV 8000 h^−1^)	141.9	61.0	43.0	[29]
2	0.1 mol % V_2_O_5_/SiO_2_			229.7	85.0	37.0	[21]
3	0.1 mol % V_2_O_5_/HMS			208.1	77.0	37.0	
4	Rb ion-modified 0.5wt% V_2_O_5_/SiO_2_			563.5	173.0	30.7	[24]
5^a^	V-Ti/MCM-41	300 W Xe with AM1.5G	C_3_H_6_/O_2_/N_2_ = 1:1:16 (GHSV 6000 h^−1^)	208.4	112.1	53.7	this study
6^b^		300 W Xe		287.3	172.1	59.9	
7^c^		200 W Hg arc		551.9	193.0	35.0	

^a^300 W Xe lamp equipped with AM1.5G filter (artificial sunlight): 1.6 mW·cm^−2^; ^b^300 W Xe lamp: 18.5 mW·cm^−2^; ^c^200 W Hg arc lamp (250–400 nm): 0.8 mW·cm^−2^. The data is the mean value obtained on stream in 6 h. V-Ti/MCM-41 photocatalyst: V/Ti/Si = 0.05:0.53:46.29, based on ICP–AES.

## Conclusion

Artificial sunlight has been successfully used to drive the photo-epoxidation of propylene for the first time, with a PO formation rate of 112.1 µmol·g_cat_^−1^·h^−1^ and a PO selectivity of 53.7% over V-Ti/MCM-41. Without AM1.5G filter, UV–visible light with higher intensity results in higher PO formation rate with a similar PO selectivity but a faster deactivation rate. UV light only with different filter (365, 320–500 and 250–400 nm, respectively) and intensity over the ranges of 0.1–0.8 mW·cm^−2^ are also examined. Among a variety of conditions, 193.0 µmol·g_cat_^−1^·h^−1^ was observed as the highest PO formation rate for propylene photo-epoxidation with a minimal deactivation rate. Data analysis suggests that the rate of PO formation and of C_3_H_6_ consumption under either UV light or UV–visible light/artificial sunlight can be correlated with NLU in log–log scale. This indicates a similar reaction mechanism under UV light and under UV–visible light/artificial sunlight, which is also supported by the same products observed under the different light source used in this study.

## Experimental

### Preparation and characterizations of photocatalyst

The procedure of V-Ti/MCM-41 preparation was described in details previously [17]. Typically, 21.2 g of sodium metasilicate monohydrate was dissolved in 100 mL deionized (DI) water and then combined with an appropriate amount of titanium oxysulfate hydrate and vanadyl sulfate hydrate (dissolved in 20 mL of 2 M H_2_SO_4_) to form a uniform gel. Next, 7.28 g of cetyltrimethylammonium bromide (CTAB) was dissolved in 25 mL of DI water and added slowly into the mixture. After stirring for 3 h, the gel mixture was transferred to an autoclave and heated to 418 K for 36 h. The resulting solid was washed with DI water after cooling to the room temperature, then dried at 383 K for 8 h, and calcined at 823 K for 10 h.

A powder X-ray diffractometer (XRD, Xray-M03XHF, Ultima IV) was used to verify the crystalline structure of the photocatalyst. Diffraction peaks were assigned by comparison to known crystalline phases. The light absorption of the photocatalyst was characterized by ultraviolet–visible light spectroscopy (Varian Cary-100). The X-ray absorption near edge spectroscopy (XANES) of the vanadium K-edge was carried out with synchrotron radiation at the beam line 16A, National Synchrotron Radiation Research Center, Taiwan. The standard metal foil and V oxides (V_2_O_5_ and V_2_O_3_) powders were used as references. High resolution transmission electron microscope (HRTEM) was performed with a JEOL JEM-2100 instrument operating at 200 kV. Thermal gravimetric analysis (TGA, PYRIS Diamond TG-DTA, high temperature 115V) was carried out in the range of 300–773 K. About 30 mg samples were placed in an alumina sample holder and heated under air (20 mL·min^−1^) with a heating rate of 3 K·min^−1^. Due to the small amount of catalyst used for the reaction, the spent is a mixture of all the photocatalysts after reaction.

### Photocatalytic epoxidation of propylene

The apparatus for carrying out the photocatalytic epoxidation of propylene with a reactant gas mixture of C_3_H_6_/O_2_/N_2_ = 1:1:16 at GHSV = 6000 h^−1^ was mentioned in our previous study [17]. Around 0.01–0.02 g of photocatalyst was packed in a photo-reactor (0.55 cm^3^ in volume) with a quartz window for light transmission. A hot-plate was used to maintain the temperature at 323 K for the UV-irradiated reaction while no heating was provided with artificial sunlight when the temperature of the photocatalyst bed was typically sustained at 312–315 K. We demonstrated previously that the photocatalytic propylene epoxidation was not sensitive to temperature in the range of 312–323 K [20]. The light sources were set up as follows: (1) **UV light:** 200 W mercury arc lamp (Exfo S1500) with three different interference filters, i.e., 365, 320–500, and 250–400 nm. The light intensity, adjustable in the range of 0.1–0.8 mW cm^−2^, was measured at the quartz window of the reactor by using a GOLDILUX radiometer/photometer (UV-A Probe/UV-C Probe). (2) **UV–visible light:** 300 W xenon lamp (Newport, USA) was directly used; the influx in the range of 200–380 nm was 18.5 mW·cm^−2^. (3) **Artificial sunlight:** A 300 W xenon lamp (Newport, USA) was used with an AM1.5G filter to simulate the sunlight that has the same power and spectral distribution of the sun at 48.5° zenith angle. The influx in the range of 310–380 nm with the AM1.5G filter was 1.6 mW·cm^−2^.

### Product analysis

The effluent stream was analyzed with a gas chromatograph (GC, Young Lin, YL6100) via an on-line 6-port sampling valve (Valco, with 1 mL loop). The GC was equipped with a flame ionization detector (FID) and a thermal conductivity detector (TCD) and the analysis was performed with both molecular sieve 5 Å and Porapak-N columns. The product formation rate, propylene consumption rate and the product selectivity were defined according to the following equations.

[6]
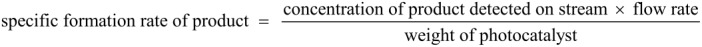


[7]
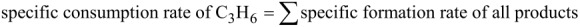


[8]